# In-Silico Functional Annotation of *Plasmodium falciparum* Hypothetical Proteins to Identify Novel Drug Targets

**DOI:** 10.3389/fgene.2022.821516

**Published:** 2022-04-04

**Authors:** Gagandeep Singh, Dinesh Gupta

**Affiliations:** Translational Bioinformatics Group, International Centre for Genetic Engineering and Biotechnology, New Delhi, India

**Keywords:** Plasmodium falciparum (3D7), hypothetical proteins, functional annotation, pathways, molecular modelling, molecular dynamcis

## Abstract

*Plasmodium falciparum* is one of the plasmodium species responsible for the majority of life-threatening malaria cases. The current antimalarial therapies are becoming less effective due to growing drug resistance, leading to the urgent requirement for alternative and more effective antimalarial drugs or vaccines. To facilitate the novel drug discovery or vaccine development efforts, recent advances in sequencing technologies provide valuable information about the whole genome of the parasite, yet a lot more needs to be deciphered due to its incomplete proteome annotation. Surprisingly, out of the 5,389 proteins currently annotated in the *Plasmodium falciparum* 3D7 strain, 1,626 proteins (∼30% data) are annotated as hypothetical proteins. In parasite genomic studies, the challenge to annotate hypothetical proteins is often ignored, which may obscure the crucial information related to the pathogenicity of the parasite. In this study, we attempt to characterize hypothetical proteins of the parasite to identify novel drug targets using a computational pipeline. The study reveals that out of the overall pool of the hypothetical proteins, 266 proteins have conserved functional signatures. Furthermore, the pathway analysis of these proteins revealed that 23 proteins have an essential role in various biochemical, signalling and metabolic pathways. Additionally, all the proteins (266) were subjected to computational structure analysis. We could successfully model 11 proteins. We validated and checked the structural stability of the models by performing molecular dynamics simulation. Interestingly, eight proteins show stable conformations, and seven proteins are specific for *Plasmodium falciparum*, based on homology analysis*.* Lastly, mapping the seven shortlisted hypothetical proteins on the *Plasmodium falciparum* protein-protein interaction network revealed 3,299 nodes and 2,750,692 edges. Our study revealed interesting functional details of seven hypothetical proteins of the parasite, which help learn more about the less-studied molecules and their interactions, providing valuable clues to unravel the role of these proteins via future experimental validation.

## Introduction

Human malaria infection, caused by the protozoa of the genus “*Plasmodium*”, is still a major public health issue, even though extensive efforts to eradicate it are in process (Uwimana et al., 2020; Hema et al., 2021). Based on a report published by the world health organization (WHO), it was estimated that 229 million cases and 409,000 deaths were reported in 2020 due to *Plasmodium falciparum* parasite alone (www.who.int/publications). Despite the best efforts and global programs regarding eliminating malaria, infection is increasing day by day due to the rapid transmission rate (Sumner et al., 2021). Although antimalarial therapies and drugs were considered helpful, growing drug resistance reduced the efficacy of drugs, which led to employing alternative methods for more effective drugs (Ramasamy et al., 2007). With the emerging sequencing technologies, various efforts were facilitated by annotating the *Plasmodium* genome (Kissinger et al., 2002; Bahl et al., 2003). Multiple studies based on proteome and genome were conducted to develop novel technologies to understand disease-resistance mechanisms in *Plasmodium* (Sardar et al., 2021; Sourabh et al., 2021). While the advancement of sequencing technologies are highly beneficial to understand specific pathways and mechanism related to the disease, the most undesirable aspect for any newly sequenced genome is when almost half of the annotated proteins or genes are in the uncharacterized category and annotated as “Hypothetical proteins (HPs)” (Galperin and Koonin, 2004; Singh et al., 2019). These uncharacterized proteins with predicted ORF regions without validated translation evidence can be categorized as “Hypothetical proteins” (Ijaq et al., 2015). Hypothetical proteins are conserved proteins and found across diverse phylogenetic lineages, thus the absence of functional annotations of the proteins is a serious concern. These proteins may be performing crucial functions, which can unravel more details of the molecular basis of the disease infection and pathogenesis (Singh and Singh, 2018).

**FIGURE 1:**
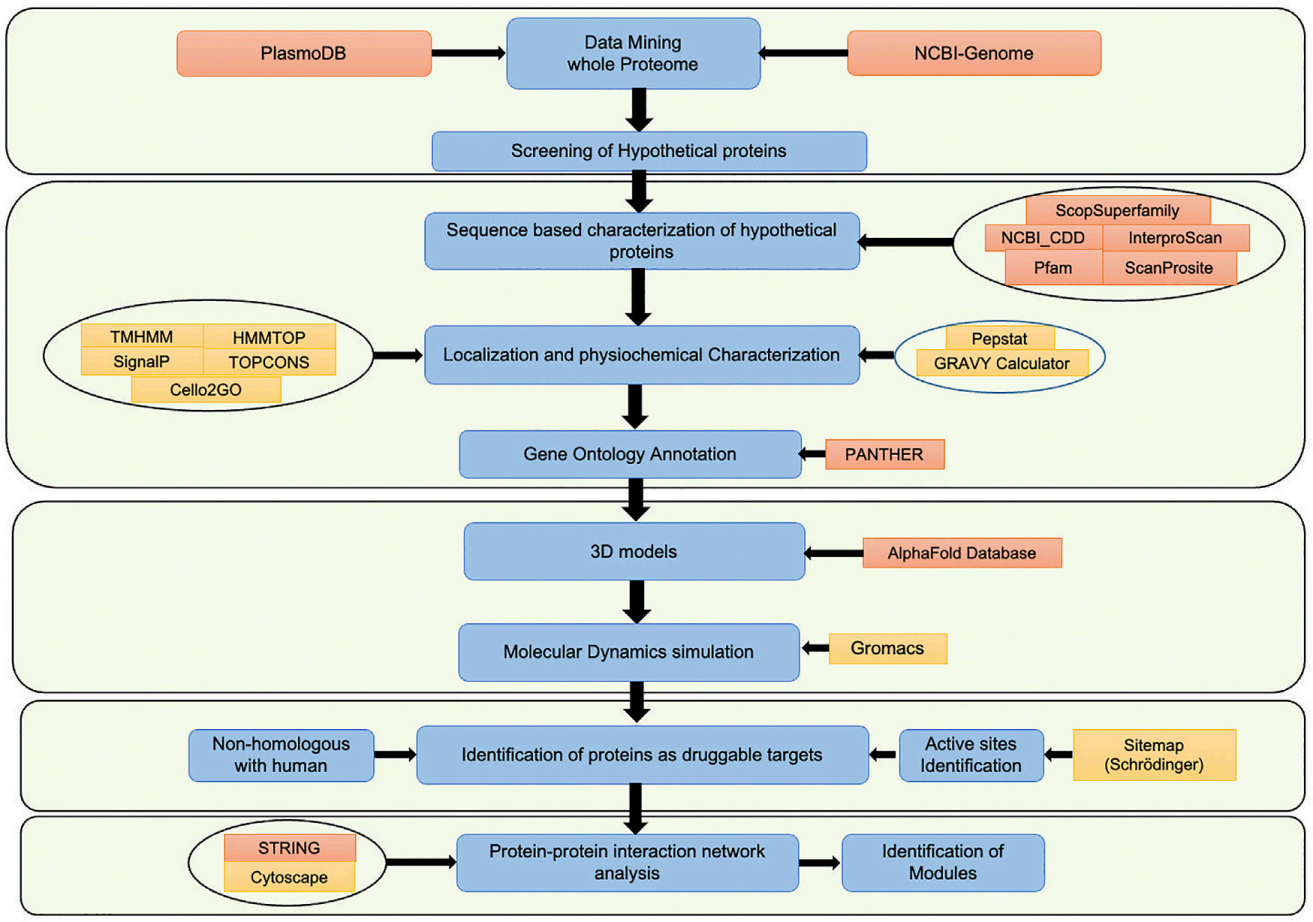
Workflow representing the overall study methodology.

In the *Plasmodium falciparum* 3D7 proteome, a total of 5,389 proteins are identified, out of which 1,626 proteins (∼30%) are hypothetical proteins (www.plasmodb.org, Bahl et al., 2003). The hypothetical proteins are often ignored in mainstream malaria research, which might have resulted in missing critical candidates for the development of malaria therapeutics. The current study has characterized HPs by following a systematic computational pipeline, based on extensive comparative analysis of sequences, results from computational structural biology, and protein-protein interaction networks (PPIs) (da Fonsêca et al., 2012; Dhanyalakshmi et al., 2016). Each computational step of the pipeline filters the best available supporting evidence to shortlist proteins with functional annotations to be a potential drug target. Our analysis revealed 266 proteins have conserved functional sites, of which structures of 11 proteins were successfully modeled and validated. After that, these proteins were mapped to a protein-protein interaction network, which revealed that these proteins interact with 3,737 other proteins. Out of 11 proteins, seven are non-homologous with human proteome and can be selected as potential targets for drug designing. Lastly, the sub-networks of each chosen protein were identified, followed by clustering and pathway enrichment analysis for further functional assessment. In the current study, we have characterized the hypothetical proteins with a view to explore them as potential novel drug targets. Also, this information can be subsequently used by malaria researchers for future experimental validation. Moreover, the study pipeline can be an effective platform to characterize hypothetical proteins in other organisms too. The workflow chart representing the overall study methodology is shown in Figure 1.

## Materials and Methods

### Data Extraction and Identification of Hypothetical Proteins

The complete proteome of *Plasmodium falciparum* 3D7 was downloaded from the PlasmoDB (https://plasmodb.org/plasmo/app, Bahl et al., 2003) and NCBI with genome reference number GCA_000002765 (https://www.ncbi.nlm.nih.gov/genome). All the sequences with functions were downloaded in fasta format and the non-redundant proteins, found to be uncharacterized or hypothetical in both the sequence datasets, were selected for further analysis. To confirm further, UniProtKB accessions of these proteins were also identified by mapping to the UniProt database (http://www.UniProt.org/HYPERLINK http://www.UniProt.org/).

### Identification of Functional Domains and Characterization of Sequences

To identify the functional signature present in the sequences of the selected hypothetical proteins, NCBI-CDD (Derbyshire et al., 2015), Interproscan (Jones et al., 2014) and SMART (Letunic et al., 2015) analysis was performed. Pfam search (Finn et al., 2014) was used to predict protein families, and for superfamilies of proteins, we employed SCOP-Superfamily assignment (Wilson et al., 2007). Furthermore, the ScanProsite tool was used to identify prosite motifs in the selected sequences (Sigrist et al., 2010). Based on the conserved domains and motifs, functions of various hypothetical proteins can be predicted at the sequence level. For higher confidence in the functional assignment, identified sequences were selected for physiochemical characterization. Theoretical isoelectric point (pI) and molecular weight were calculated by using Compute pI/Mw tool (Bjellqvist et al., 1994). The values for the grand average of hydropathy of protein sequences were examined using GRAVY CALCULATOR (http://www.gravy-calculator.de/). Additionally, to evaluate the aromatic and aliphatic properties of the sequences with the average number of polar and non-polar amino acids, the acidic and basic nature of protein sequences, the EMBOSS PepStat tool was used (Rice et al., 2000).

### Protein Localization, Functional Annotation and Pathway Analysis

After identifying functional sequence signatures in the hypothetical proteins, we analyzed the proteins’ predicted cellular and sub-cellular localization. Predicted cellular localization were determined using TMHMM (Krogh et al., 2001), HMMTOP (Tusnady and Simon, 2001) and TOPCONS (Tsirigos et al., 2015). Whereas the sub-cellular location was predicted using CELLO2GO (Yu et al., 2014) and signal peptide prediction using SignalP (Emanuelsson et al., 2007). For discovering the functional roles of these proteins, gene ontology was predicted using PANTHER (Mi et al., 2021). This tool is used to predict proteins’ cellular components, biological processes, and molecular functions. Using the KEGG database, the role of hypothetical proteins in different pathways was analyzed (Kanehisa and Goto, 2000).

### Structural Analysis and Validation of Proteins

The structures of the shortlisted proteins were obtained from the AlphaFold protein structure database (https://alphafold.ebi.ac.uk). The overall quality of the selected structures were validated using SAVES (Structure Analysis and Verification Server) by estimating stereochemical quality at the molecular level, parameters of residues, model compatibility, non-bonded interactions, and macromolecular volume of atoms (Lüthy et al., 1992; Colovos and Yeates, 1993; Hooft et al., 1996; Pontius et al., 1996). To check the residues in the most favored regions of the Ramachandran plot, PROCHECK at the SAVES server was used (Pontius et al., 1996).

### Molecular Dynamics Simulation of Proteins

To check the stability of validated structures, MD simulations of the models were performed using GROMACS 5.0 (GROningen MAchine for Chemical Simulation) package, using the CHARMM27 force field and files related to the topology of proteins generated using the pdb2gmx command. Firstly, the proteins were solvated and placed in a cubic box with a distance of 1 nm between box edges and the surface of proteins (Berendsen et al., 1995). As per the requirement of proteins, periodic boundary conditions and PME electrostatics were applied in all directions and Na^+^ ions were added to neutralize the systems. To avoid the steric clashes within the system, energy minimization was carried out using 50,000 steps of steepest descent. For MD simulations, each system was equilibrated (at NVT and NPT) for 10ns at 300 K temperature and a pressure of 1 bar. Sampling was carried out at 10ps intervals during MD production (RA and MB, 2011).

### Homology Analysis of Proteins With Humans, Followed by Active Site Pockets Identification

Sequences of the structurally validated proteins were compared with the human proteome sequence data (human; taxid: 9,906) to identify non-homologous proteins using BLASTP (Altschul et al., 1997). Proteins hits with E-value (expectation value) less than 0.0001 were considered to be in the homologous category and hence cannot be selected for drug designing, while non-homologous proteins can be regarded as to be specific for *Plasmodium falciparum* 3D7. The homology of the proteins was also checked using OrthoMCL analysis in PlasmoDB. Finally, the identified proteins were subjected to active site identification using Schrödinger’s “sitemap” (Jacobson et al., 2004).

### Protein-Protein Sub-Network Analysis of Characterized Proteins

The proteome-wide interaction network of *Plasmodium falciparum* was downloaded from the STRING (v11.5) database (Szklarczyk et al., 2021). Functionally characterized proteins were mapped to this network and their first interacting nodes were identified to construct a sub-network of targeted proteins, using Cytoscape software 3.8.2 (Shannon et al., 2003). Further to identify top modules for each of the characterized proteins, MCODE clustering was performed by separately selecting the first neighbors of each protein. After that, pathway enrichment analysis of each protein’s top modules was examined using the KEGG database (Kanehisa and Goto, 2000) and DAVID Bioinformatics Resources 6.8 (Jiao et al., 2012).

## Results and Discussion

### Identification of Hypothetical Proteins and Sequence-Based Analysis

Currently, 5,389 proteins are annotated in the whole proteome of *Plasmodium falciparum* 3D7 available in the PlasmoDB resource (release 53, https://plasmodb.org/plasmo/app, Bahl et al., 2003) and NCBI genome (reference number GCA_000002765, https://www.ncbi.nlm.nih.gov/genome). Sequence-based analysis revealed that 1,608 proteins (∼30%) are annotated as hypothetical proteins, which were selected for functional characterization, using downstream steps in the study pipeline. The existence of these proteins was further confirmed by mapping with the UniProt database (http://www.UniProt.org/) and UniProtKB accessions were identified. Out of the 1,608 proteins, 266 proteins were found to have conserved motifs, which can provide helpful information about functional sites and predict their functions. Further analysis of the proteins revealed 133 proteins are represented by specific families, and 127 proteins have a specific superfamily. Furthermore, the physicochemical characterization revealed that 84 proteins have acidic nature (PI < 7) and 182 proteins are found to be basic (Singh et al., 2019). Moreover, the aliphatic index of these proteins (266 proteins) was found to be in the range of 8.5–34.7. To check the stability of proteins at a wide range of temperatures, a higher aliphatic index is considered as positive for their stability at higher temperatures (Singh et al., 2017). GRAVY index (GI) (hydrophobicity criteria of a typical protein) was found in the range of −2.016 to 0.72 for all the proteins with conserved functional sites. The role of GI is to determine the interaction of proteins with water molecules, where the positive value of GI determines hydrophobic nature and negative values of GI determine the hydrophilic nature of the protein (Zhang et al., 2006). Predicted functions of the proteins can be reexamined on the basis of physicochemical characterization, for a particular environment of protein existence (Singh et al., 2017). Details of proteins with functional sites and physiochemical characterization are shown in Supplementary Table S1.

### Cellular Localization, Pathway Analysis and Functional Annotation of Proteins

Based on prediction of cellular localization, the biological function of the proteins can be elucidated as this is an important criterion to define the functions in a specific environment (Yu et al., 2006). Also, assigned functions of specific proteins can be assessed for cell-specific localization and their important regulatory roles. A total of 115 proteins were predicted to be present in the transmembrane and 155 proteins were cytosolic. Additionally, subcellular localization prediction revealed that 76.7% of proteins are present in nuclear regions, followed by plasma membrane (16.7%) and mitochondrial regions (4.9%). Of these, 18 proteins were found to have signal peptides as well. Signal peptides have an essential role in carrying information related to protein secretion, disease diagnosis and immunization processes (Owji et al., 2018). Details related to cellular, sub-cellular localization and signal peptides are shown in Supplementary Table S2.

**FIGURE 2:**
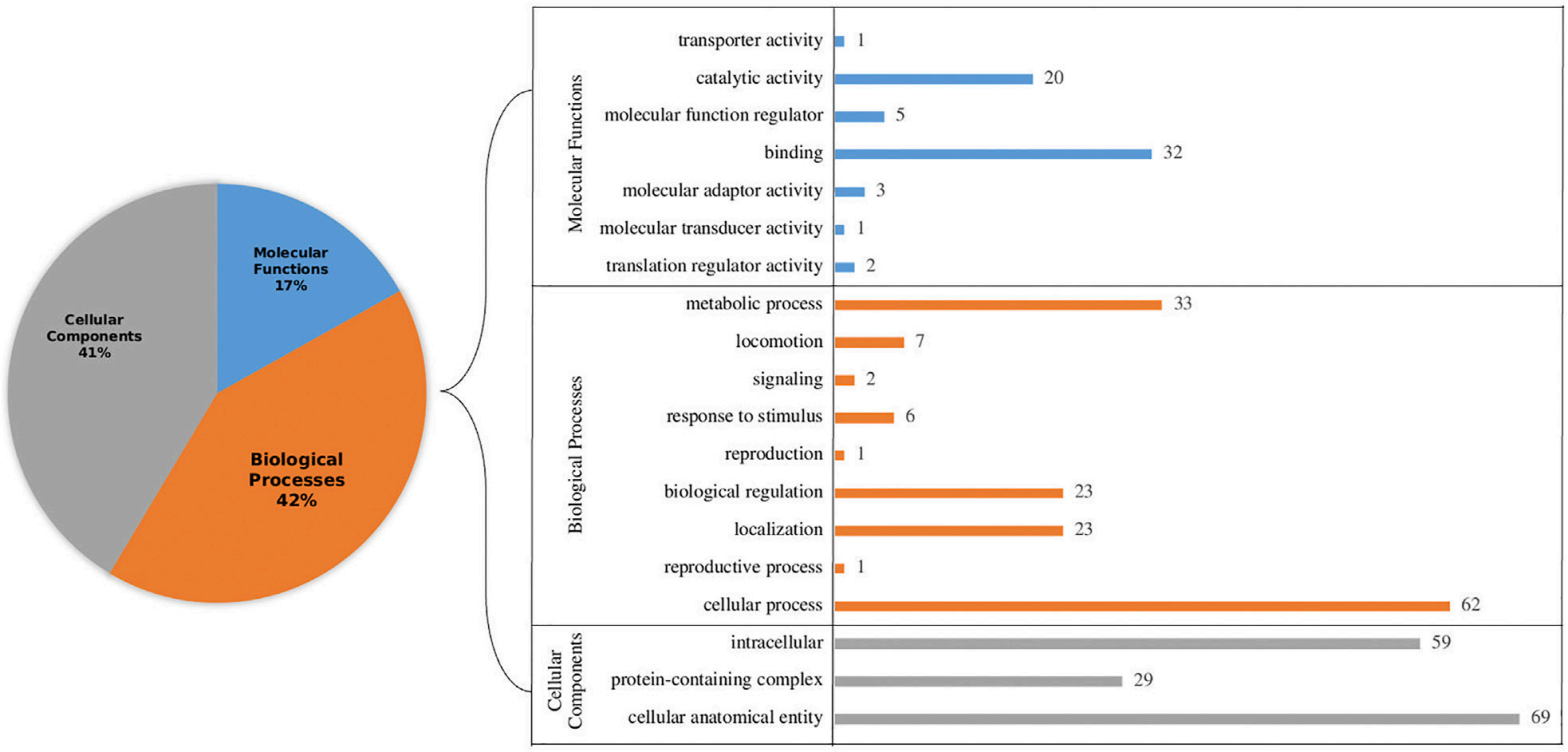
Gene Ontology (GO) annotation of hypothetical proteins with functional signature sites present in the primary sequences to identify functional categories: cellular components, biological processes and molecular functions level. The left panel shows a pie chart of the overall percentage. The right panel shows the precise breakup of sub-components in each category.

Further, all the selected proteins (266) were subjected to gene ontology annotations. Among several categories, the largest cluster was cellular processes followed by metabolic processes in the biological processes, while cellular anatomical entity and intercellular were highest in cellular components. Likewise, among molecular functions, binding and catalytic activity were most abundant. Representation of Gene Ontology annotation for all the categories is shown in Figure 2. Furthermore, pathway analysis revealed that 23 proteins have an essential role in particular pathways as well. These pathways include metabolism activities, genetic information processing and cellular processes (Supplementary Table S2). Understanding metabolic activities is essential for designing inhibitors targeting crucial metabolic activities, which can potentially lead to the death of the parasites using antimalarial compounds (Calas et al., 2000). To unravel the regulatory machinery of an organism, such as gene regulation, it is crucial to find all the missing links which might be present in the hypothetical category. Genetic information processing has a vital role in understanding gene regulatory mechanisms (Kudyba et al., 2021). Pathways related to cellular processes such as ‘transport and catabolism’ were also predicted. However, it may be noted that although studies based on comparative and functional genomics of *Plasmodium falciparum* revealed several important functions and mechanisms, cellular functions are not clear yet (Le Roch et al., 2012). By means of cellular processes, the TCA cycle and other important flux mechanisms can be identified so that inhibitors can be designed to cure malaria (Ke et al., 2015).

### Structural Analysis of Characterized Proteins

**FIGURE 3:**
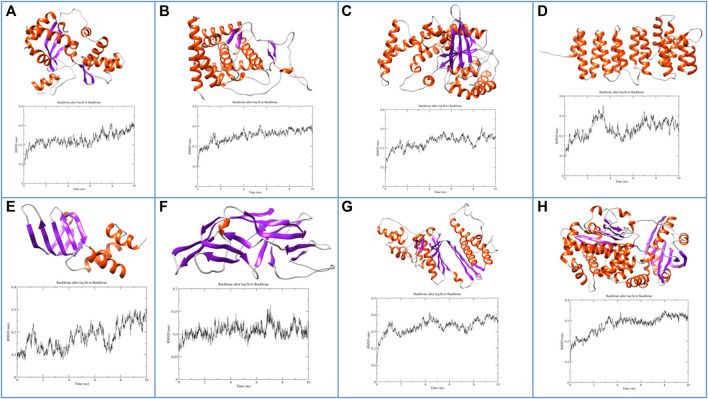
Molecular structures and MD trajectory RMSD graphs of the shortlisted proteins **(A)** PF3D7_1368300, **(B)** PF3D7_1204500, **(C)** PF3D7_1308500, **(D)** PF3D7_1438600, **(E)** PF3D7_0418400, **(F)** PF3D7_1351100, **(G)** PF3D7_1442800 and **(H)** PF3D7_1402000.

After sequence-based functional analysis, all the selected proteins (266) were subjected to computational structural analysis. Based on the Alphafold protein structure database, 11 proteins passed validation based on Ramachandran plot analysis. Also, functional assignments, based on sequence analysis, are the same as identified at the structural level, further confirming the accuracy of functional assignments of the respective hypothetical proteins. Additionally, to check the stability of these proteins, energy minimization and MD simulations revealed eight proteins to be structurally stable as revealed by analysis of the parameters such as radius of gyration, solvent accessible surface area and root-mean-square fluctuations. Structures and RMSF graphs of all the stable proteins are shown in Figure 3 and Ramachandran plots of respective structures are shown in Supplementary Figure S1. Protein PF3D7_1368300 has been annotated as “Non-structural maintenance of chromosome element 1 (Nse1)”. The Smc5/6 complex has a crucial role in chromosome replication and repairing DNA, within this, a sub-complex of Nse1, Nse3 and Nse4 might have multiple roles by DNA binding and regulation of ATP-dependent activities of the complex (Jo et al., 2021). Nse1 has a RING finger identical to E3 ubiquitin ligases with a crucial role in DNA repair processes and resolving recombination structures of chromosomes (Taylor et al., 2008). Protein PF3D7_0418400 belongs to the Sm-like (Lsm) family of proteins with an essential role in RNA metabolism (Scofield and Lynch, 2008). Lsm proteins have a crucial role in mRNA processing, telomere elongation and ribosomal assembly (Fernandéz-Taboada et al., 2010). Protein PF3D7_1204500 is encoded as a small nuclear RNA activating complex (SNAPc) subunit SNAP43. This protein has an essential role in the functioning of the spliceosome, as already reported in *Plasmodium falciparum* (Horrocks et al., 2009). Also, it contains a variable amount of TATA box-binding proteins (TBP) and is required by RNA polymerase II and III for the transcription of snRNA genes (Sadowski et al., 1996). Protein PF3D7_1308500 is annotated as P-loop containing nucleoside triphosphate hydrolase, a large family of proteins with diverse cellular functions. P-loop proteins can be characterized by a conserved pattern of sequence GXXXXGKS, known as Walker A motif, also found in the protein PF3D7_1308500, annotated in our study as well (Pathak et al., 2014). Protein PF3D7_1442800 is identified as translation elongation factor (EF-Ts), with an important role in catalysing nucleotide exchange in elongation factor Tu (EF-Tu) and promoting the formation of EF-Tu. GTP from EF-Tu. GDP (Wieden et al., 2002). Another study revealed that EF-Ts, along with EF-Tu, EF-G1 and release factor RF1, impairs growth and oxidative phosphorylation (Cristodero et al., 2013). Protein PF3D7_1438600 is annotated as Golgi to ER traffic protein 4 (Get4), a tail-anchored (TA) protein with multiple roles such as response to stress and electron transport. Get4 forms a hetero-tetrameric complex along with Get5 and mediates delivery of tail-anchored (TA) substrates from Sgt2 (small glutamine-rich, tetratricopeptide repeat protein 2) to Get3 (Chang et al., 2010; Chartron et al., 2010). The Protein PF3D7_1402000 belongs to the DHH superfamily of proteins, consisting of a conserved triad motif DHH (Asp-His-His) that performs a vital role as phosphoesterases and phosphatases. The motif (DHH) is also present in PF3D7_1402000, a shortlisted protein. These proteins have diverse functions, ranging from DNA repair, nucleic acid metabolism, maintenance of stress conditions, etc. (Srivastav et al., 2019).

### Identification of Proteins as Drug Designing Targets

**FIGURE 4:**
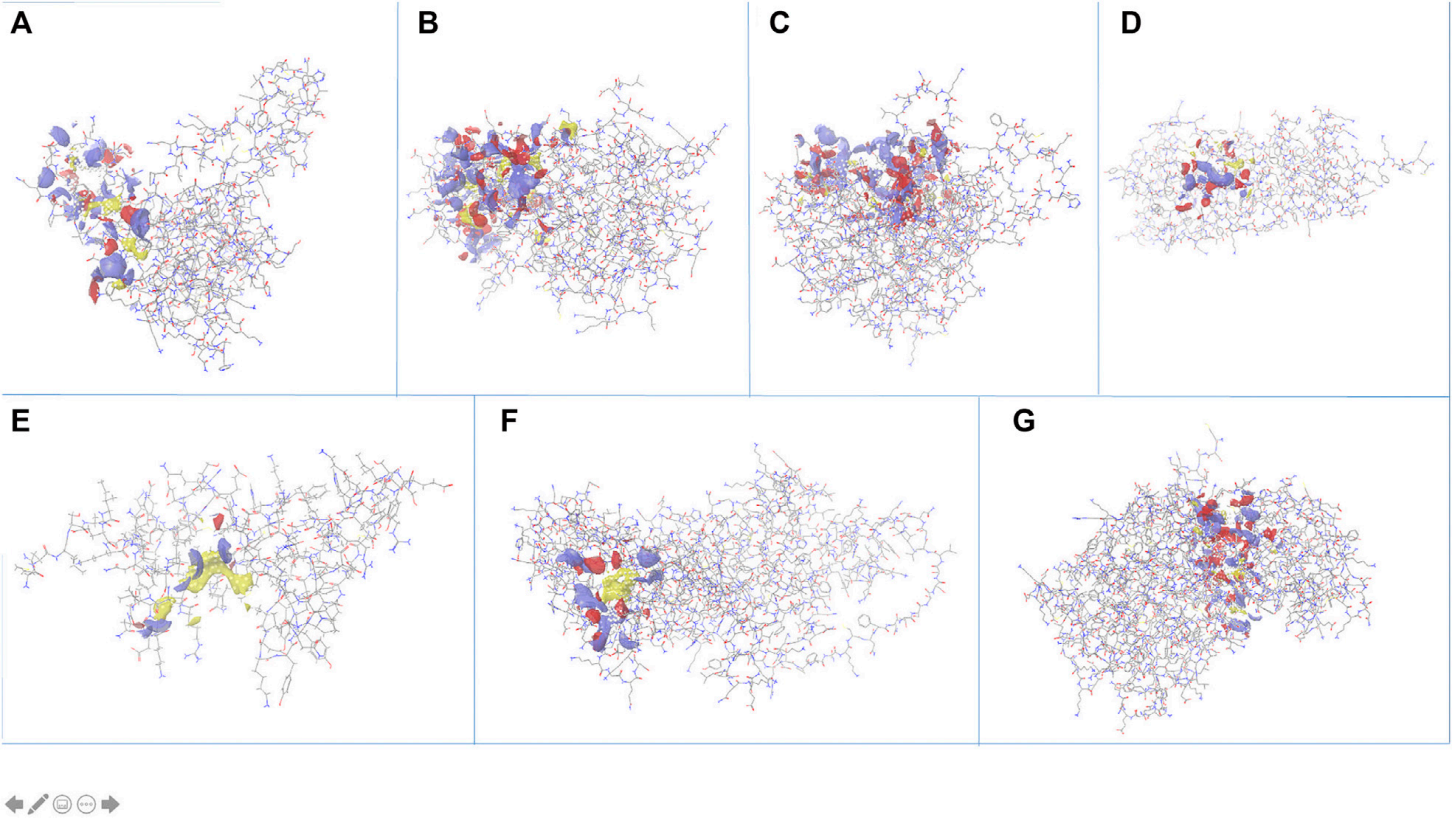
Identification of active site pockets (highlighted as clouds) for the shortlisted proteins **(A)** PF3D7_1368300, **(B)** PF3D7_1204500, **(C)** PF3D7_1308500, **(D)** PF3D7_1438600, **(E)** PF3D7_0418400, **(F)** PF3D7_1442800 and **(G)** PF3D7_1402000.

The search for non-homologous proteins, by searching sequence similarity with host proteome (taxid:9,606), helped filter out the best suitable candidate targets for drug designing. Out of the eight stable proteins, seven are *Plasmodium falciparum* specific, also confirmed by OrthoMCL, that may be explored for new drug designing strategies. All the proteins were subjected to identifying active sites using “sitemap” in the Schrödinger package. After pre-processing, the top-ranked potential receptor binding sites and respective residue were identified based on SiteScore. All the sites are shown in Figure 4 and a list of respective residues are provided in Supplementary Table S2. The proteins with top ranked binding sites can be used to design effective lead molecules as inhibitors, through virtual screening, targeting these sites.

### Protein-Protein Interaction Network and Pathway Enrichment Analysis

**FIGURE 5:**
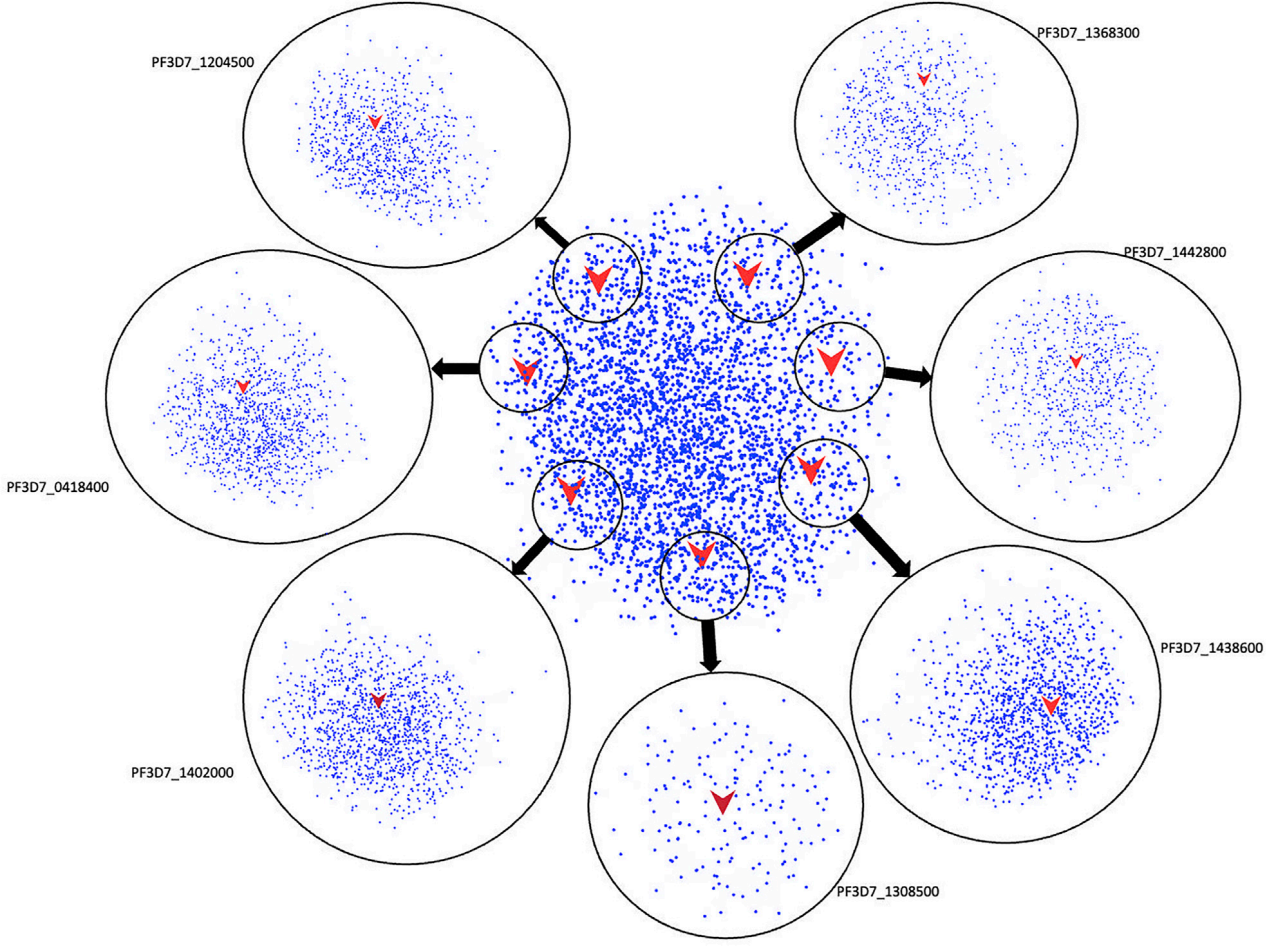
Protein-Protein interaction networks: The overall Protein-Protein interaction network of the proteins selected as drug targets in the center and individual sub-networks of each protein with interacting nodes zoomed out as circles.

The previously determined protein-protein interaction network of *Plasmodium falciparum* 3D7 was explored to find the key regulatory proteins involved in the various important activities such as metabolism and immune system mechanisms (Saha et al., 2018). All the structurally characterized proteins (11 proteins) interacted with 3,737 other proteins, as shown in Figure 5. Further proteins that were stable and specific to *Plasmodium falciparum* (7 Proteins) were subjected to sub-network analysis (Figure 5), followed by clustering and top modules were selected for pathway enrichment analysis. Interestingly, the protein PF3D7_1368300 is interacting with 632 other proteins, and by clustering analysis, it is found that 187 proteins are present in the top ranked module. Oxidative phosphorylation in energy metabolism is highly enriched, followed by replication and repair mechanism, which further affirms the assigned function with a role in chromosome replication and repairing DNA (Jo et al., 2021). A total of 1,069 nodes interact with the protein PF3D7_0418400 and in the top module, 449 proteins are found after clustering analysis. Translation activity and spliceosome activity are highly enriched, followed by proteosome and RNA degradation, as evident from pathway enrichment analysis. Network analysis further confirmed the role of protein PF3D7_0418400 as mRNA processing, telomere elongation and ribosomal assembly (Fernandéz-Taboada et al., 2010). Protein PF3D7_1204500 is interacting with 838 nodes and a total of 280 proteins are found to be present in the top module. The replication and repair mechanism is highly enriched, followed by translation revealed by the pathway enrichment analysis, which further confirmed the assigned function of the protein PF3D7_1204500. Protein PF3D7_1308500 is connected with 149 nodes and clustering reveals that 118 proteins are present in its top module. Pathway enrichment analysis revealed that DNA replication is highly enriched, followed by nucleotide excision repair. The predicted role of PF3D7_1308500 protein is P-loop containing nucleoside triphosphate hydrolase, which is a large family of proteins with diverse cellular functions, including replication and repair mechanisms (Pathak et al., 2014). Protein PF3D7_1442800 has 663 first nodes in the PPI subnetwork and 111 proteins are present in the top module. Pathway enrichment reveals that basal transcription factors and spliceosome are highly enriched, followed by metabolic pathways. Analysis based on the PPI network further indicates the role of this protein in growth and oxidative phosphorylation, as predicted by sequence analysis. PF3D7_1438600 is interacting with 1,137 other proteins, with 647 proteins found in the top module. Translational pathways such as Ribosomal, Aminoacyl-tRNA biosynthesis, Nucleocytoplasmic transport are highly enriched, again its assigned function is confirmed at the sequence level. Protein PF3D7_1402000 is identified to interact with 1,211 nodes and clustering revealed 382 nodes in its top ranked module. Ribosome activity is highly enriched in the translation pathway, followed by spliceosome and biosynthesis of secondary metabolism.

## Conclusion

With the continuous revolutionization of next generation sequencing technologies, large-scale genomic or transcriptome data of several organisms can be generated in a single run. Although annotation methods for the analysis of generated data are available on a large scale, the most disconcerting aspect is that half of the generated data remains uncharacterized and comes under the “Hypothetical category”. Functional assignment of the hypothetical proteins can be elucidated experimentally, however, experimental validations are time-consuming, expensive and in several cases, technically not feasible. In this current study, multifaceted approaches such as domain-based characterization and physiochemical characterization on the basis of primary sequence analysis revealed the functions of 266 *P. falciparum* proteins. Further, the functions of 11 proteins were successfully validated by *in-silico* structural analysis.

Furthermore, protein-protein interaction revealed that these 11 proteins are interacting with 3,737 other *Plasmodium falciparum* proteins*.* After that, molecular dynamics simulation revealed that eight proteins are stable. Of these, seven proteins are specific to *Plasmodium falciparum* which can be explored for their essentiality in the parasite and design lead molecules as inhibitors. The methodology followed in this study can also be extrapolated to assign functions to hypothetical proteins in other organisms too.

## Data Availability

Publicly available datasets were analyzed in this study. This data can be found here: www.plasmodb.org.
